# Neoadjuvant chemoradiation followed by surgery versus surgery alone for patients with adenocarcinoma or squamous cell carcinoma of the esophagus (CROSS)

**DOI:** 10.1186/1471-2482-8-21

**Published:** 2008-11-26

**Authors:** M van Heijl, JJB van Lanschot, LB Koppert, MI van Berge Henegouwen, K Muller, EW Steyerberg, H van Dekken, BPL Wijnhoven, HW Tilanus, DJ Richel, ORC Busch, JF Bartelsman, CCE Koning, GJ Offerhaus, A van der Gaast

**Affiliations:** 1 Department of Surgery, Academic Medical Center, Amsterdam, The Netherlands; 2 Department of Surgery, Erasmus Medical Center, Rotterdam, The Netherlands; 3 Department of Radiotherapy-Oncology, Erasmus Medical Center, Rotterdam, The Netherlands; 4 Department of Clinical Epidemiology, Erasmus Medical Center, Rotterdam, The Netherlands; 5 Department of Pathology, Erasmus Medical Center, Rotterdam, The Netherlands; 6 Department of Medical Oncology, Academic Medical Center, Amsterdam, The Netherlands; 7 Department of Gastroenterology, Academic Medical Center, Amsterdam, The Netherlands; 8 Department of Radiotherapy, Academic Medical Center, Amsterdam, The Netherlands; 9 Department of Pathology, Academic Medical Center, Amsterdam, The Netherlands; 10 Department of Medical Oncology, Erasmus Medical Center, Rotterdam, The Netherlands

## Abstract

**Background:**

A surgical resection is currently the preferred treatment for esophageal cancer if the tumor is considered to be resectable without evidence of distant metastases (cT1-3 N0-1 M0). A high percentage of irradical resections is reported in studies using neoadjuvant chemotherapy followed by surgery versus surgery alone and in trials in which patients are treated with surgery alone. Improvement of locoregional control by using neoadjuvant chemoradiotherapy might therefore improve the prognosis in these patients. We previously reported that after neoadjuvant chemoradiotherapy with weekly administrations of Carboplatin and Paclitaxel combined with concurrent radiotherapy nearly always a complete R0-resection could be performed. The concept that this neoadjuvant chemoradiotherapy regimen improves overall survival has, however, to be proven in a randomized phase III trial.

**Methods/design:**

The CROSS trial is a multicenter, randomized phase III, clinical trial. The study compares neoadjuvant chemoradiotherapy followed by surgery with surgery alone in patients with potentially curable esophageal cancer, with inclusion of 175 patients per arm.

The objectives of the CROSS trial are to compare median survival rates and quality of life (before, during and after treatment), pathological responses, progression free survival, the number of R0 resections, treatment toxicity and costs between patients treated with neoadjuvant chemoradiotherapy followed by surgery with surgery alone for surgically resectable esophageal adenocarcinoma or squamous cell carcinoma. Over a 5 week period concurrent chemoradiotherapy will be applied on an outpatient basis. Paclitaxel (50 mg/m2) and Carboplatin (Area-Under-Curve = 2) are administered by i.v. infusion on days 1, 8, 15, 22, and 29. External beam radiation with a total dose of 41.4 Gy is given in 23 fractions of 1.8 Gy, 5 fractions a week. After completion of the protocol, patients will be followed up every 3 months for the first year, every 6 months for the second year, and then at the end of each year until 5 years after treatment. Quality of life questionnaires will be filled out during the first year of follow-up.

**Discussion:**

This study will contribute to the evidence on any benefits of neoadjuvant treatment in esophageal cancer patients using a promising chemoradiotherapy regimen.

**Trial registration:**

ISRCTN80832026

## Background

Esophageal cancer is a highly lethal disease, as reflected by an overall 5-year survival rate of 10%.[1] With worldwide almost 400,000 new patients diagnosed annually, esophageal cancer is the eighth most common cancer, and sixth on the list of cancer mortality causes.[2] The total incidence of esophageal cancer is rising, mainly as the result of a marked rise in the incidence of adenocarcinoma.[3]

Surgical resection is currently the preferred treatment for esophageal cancer if a patient is fit enough to undergo major surgery and the tumor is considered to be resectable without evidence of distant metastases (cT1-3 N0-1 M0). However, approximately 30% of operated patients, clinically considered to have resectable disease, have microscopically irradical resections performed on. [4-6]

The goals of neoadjuvant chemotherapy are a reduction of recurrence from occult lymphatic and/or distant metastases with improvement of survival and possible tumor shrinkage with an increased radical resectability rate. In many of the performed phase II studies the patients who had objective response to chemotherapy had a significantly better survival compared to non-responding patients.[7,8]

The number of randomized phase III studies comparing neoadjuvant chemotherapy followed by surgery versus surgery alone is limited. [5-13] The results of these randomized phase III studies and the results of reviews show that the possible benefit, if any, of neoadjuvant-chemotherapy for patients with esophageal cancer is small. It is uncertain whether such a small potential survival benefit outweighs the morbidity caused by such a treatment.[14,15] A surgery only arm is therefore still considered to be appropriate in randomized phase III studies for patients with esophageal cancer.

Chemotherapy and radiotherapy can interact in several ways. Both treatment modalities may be active against different tumor cell populations (additive effect), the chemotherapy may be effective against micrometastases while radiation is active locoregionally ("spatial cooperation"). Chemotherapy may synchronize cells in a vulnerable phase for radiotherapy, decrease repopulation after radiotherapy and, by shrinking a tumor, enhance reoxygenation, which is advantageous for radiotherapy.[16,17]

In an Intergroup trial (INT 0123 – RTOG 94-05) patients were randomized to receive the chemoradiotherapy regimen as was used in the RTOG 85-01 trial (with 50 Gy radiotherapy) or the same chemotherapy regimen combined with 64.8 Gy radiotherapy.[18,19] After an interim analysis the trial was closed prematurely because of a high number of treatment related deaths in the high-dose radiotherapy arm. There was no significant difference in median or 2-year survival between the two arms. The EORTC reported on a prospective randomized study of split-course radiotherapy (2 × 20 Gy, 4 Gy each fraction, 5 fractions a week with a 2 week gap) with or without 2 courses cisplatin 100 mg/m^2 ^given 3 or 4 days before the start of radiotherapy and 4 courses afterwards.[20] The median overall survival was 7.9 months for patients in the radiotherapy arm en 9.6 months for the patients treated with chemoradiotherapy. There was no significant difference in overall survival. Median time to local progression was 6.2 months against 10.9 months in favor of the chemoradiotherapy arm. This split course radiotherapy and timing of chemotherapy may be not considered optimal nowadays.

These studies show that concurrent chemoradiation is recommended compared to radiotherapy alone. Furthermore, higher dose of radiotherapy (50 versus 64 Gy) in combination with chemotherapy will increase toxicity rates with no difference in survival.

In most concurrent chemoradiotherapy studies the classic 5-fluorouracil – cisplatin regimen has been used. More recently, studies with taxanes as concurrently administered cytotoxic drugs showed promising results. The combination of Paclitaxel and Carboplatin with concurrent radiotherapy has been successfully applied in patients with advanced non-small cell lung cancer. In 3 studies the combination of Paclitaxel and Carboplatin was given weekly with concurrent chest radiotherapy followed by 2 21-day cycles of consolidation chemotherapy. [21-23] These studies reported no unexpected toxicity, and esophagitis was the most frequent severe toxicity. The response rates were high and the early survival results were encouraging. Another approach was two induction cycles of Paclitaxel and Carboplatin prior to chest radiotherapy with concurrent chemotherapy of Paclitaxel and Carboplatin [24,25] or followed by 2 additional chemotherapy cycles.[26] These studies reported lower response rates than the first approach, however, survival was similar. Awaiting conclusive randomized trials the combination of Paclitaxel, Carboplatin and radiation is a promising treatment for locally advanced non-small cell lung cancer with high response rates and acceptable toxicity.

A few years ago, a phase II study was performed in Rotterdam.[27] In this study patients with resectable adenocarcinoma or squamous cell carcinoma of the esophagus were treated with neoadjuvant concurrent chemoradiation. Patients were treated with 5 weekly cycles of Paclitaxel (50 mg/m^2^) and Carboplatin (AUC = 2) in combination with 41.4 Gy radiotherapy given in 23 fractions. Forty patients have completed treatment as per protocol. All patients received 100% of the planned dose of chemotherapy and radiotherapy. Myelotoxicity was mild and no infectious complications were observed. Esophagitis was the predominant toxicity and three patients required tube feeding, however, all these patients had already a severely impaired passage for food at the start of treatment. All but one patient had a radical surgical resection (R0-resection). This compares favorably with the radical resection rate in other studies with or without neoadjuvant chemotherapy.[5,6] Many patients had either a complete pathological response or only small tumor remnants after completion of the chemoradiation and all but one had negative lymph nodes.

A high percentage of irradical resections is reported in studies using surgery alone or neoadjuvant chemotherapy followed by surgery.[5,6,28] It is thus obvious that adequate locoregional control is still an important issue in the treatment of patients with esophageal cancer. Improvement of loco-regional control by using neoadjuvant chemoradiotherapy might therefore improve the prognosis in these patients. The results of our phase II study with neoadjuvant chemoradiotherapy with weekly administrations of Carboplatin and Paclitaxel combined with concurrent radiotherapy show that with this regimen nearly always a complete R0-resection can be performed.[27] The concept that neoadjuvant chemoradiotherapy improves the overall survival has, however, to be proven in a randomized phase III trial.

## Methods/design

### Study objectives

The objective of the CROSS trial is to compare median survival rates and quality of life (before, during and after treatment) between patients treated with neoadjuvant chemoradiotherapy followed by surgery with surgery alone for surgically resectable esophageal adenocarcinoma or squamous cell carcinoma.

As secondary objectives we aim to compare pathological responses, progression free survival, the number of R0 resections, treatment toxicity and costs.

### Study design

The CROSS trial is a multicenter, randomized phase III, clinical trial. The study started on 1 January 2004 and the duration of inclusion will be approximately 5 years. The study compares neoadjuvant chemoradiotherapy followed by surgery with surgery alone in patients with potentially curable esophageal cancer, with inclusion of 175 patients per arm.

### Patient selection

Patients with histologically proven squamous cell carcinoma or adenocarcinoma of the esophagus or gastro-esophageal junction will undergo extensive preoperative staging, including endosonography and spiral CT-scan of the chest and abdomen. The tumor must not extend more than 2 cm into the gastric cardia. Longitudinal tumor length must not exceed 8 cm, radial size must not exceed 5 cm. cT1N0 tumors are not eligible. Patients must have adequate hematological, renal, hepatic and pulmonary functions defined as: granulocytes ≥ 1.5 × 10^9^/L, platelets ≥ 100 × 10^9^/L, total bilirubin ≤ 1.5 × upper normal limit, creatinine ≤ 120 μmol/L and FEV1 ≥ 1.5 L. In the absence of local irresectability and/or distant dissemination patients with an acceptable general condition (ECOG performance status 0, 1, 2; weight loss < 10%) will be invited to participate in the randomized trial. After written, voluntary, informed consent and stratification for tumor type, treatment center, clinical lymph node status, and WHO performance status the patients will be randomized between the two treatment-arms (neoadjuvant chemoradiation followed by surgery versus surgery alone).

### Chemotherapy regimen

Paclitaxel 50 mg/m^2 ^and Carboplatin AUC = 2 will be given by intravenous infusion on days 1, 8, 15, 22 and 29. All patients receiving Paclitaxel will receive half an hour before the start of the Paclitaxel infusion premedication: Dexamethason 10 mg i.v., Clemastine (Tavegil) 2 mg i.v. and Ranitidine (Zantac) 50 mg i.v..

At hour 0, the total calculated dose of Paclitaxel, diluted in 500 ml of normal saline will be infused over one hour. After the completion of the Paclitaxel infusion, 100 ml NaCl 0.9% will be infused over 0.5 h, followed by an infusion of 8 mg Ondansetron or its equivalent diluted in 100 ml NaCl 0.9% over 0.5 hour. Hereafter the total calculated dose of Carboplatin, diluted in 500 ml glucose 5% will be infused over one hour (doses Carboplatin > 250 mg should be dissolved in 1000 ml glucose 5%). The absolute dose of Carboplatin will be calculated for the target AUC = 2 according to the following formula: the absolute dose of Carboplatin = [target AUC] × (Glomerular Filtration Rate + 25).

It is possible that some patients will experience asymptomatic bradycardia during the Paclitaxel infusion. In addition, hypersensitivity reactions are possible and generally occur within the first few minutes of initiating the infusion. For these reasons, it is recommended that there is constant supervision and that the vital signs are monitored every fifteen minutes during Paclitaxel administration. Thereafter, patients may be observed and heart rate and blood pressure checked if necessary, according to clinical symptoms.

### Radiotherapy treatment

A total dose of 41.4 Gy will be given in 23 fractions of 1.8 Gy, 5 fractions per week, starting the first day of the first cycle of chemotherapy. All patients will be radiated by external beam radiation, using 3-D conformal radiation technique. The patient will be positioned in supine position. Reproducibility will be assessed by orthogonal laser beams.

The Gross Tumor Volume (GTV) is defined by the primary tumor and any enlarged regional lymph nodes.[29], and will be drawn on each relevant CT slice. The GTV will be determined using all available information (physical examination, endoscopy, EUS, CT-thorax/abdomen).

The Planning Target Volume (PTV) will provide a proximal and distal margin of 4 cm, in case of tumor extension into the stomach, a distal margin of 3 cm will be chosen. A 2 cm radial margin around the GTV will be provided to include the area of subclinical involvement around the GTV and to compensate for tumor motion and set-up variations.

Both lungs will be contoured. The heart will be contoured on all slices; its cranial border will include the infundibulum of the right ventricle and the apex of both atria.[30], and will exclude the great vessels as much as possible. The caudal border will be defined as the lowest part of the left ventricle's inferior wall that is distinguishable from the liver. The spinal canal will be contoured and taken to represent the spinal cord. Prior to the start of the irradiation a planning CT scan will be made from the cricoid to L1 vertebra with a slice thickness of 5 mm, with the patient in treatment position. The isocenter will be determined at the planning-CT.

Radiation therapy will be delivered using a multiple field technique. Treatment can be given with the combination of anterior/posterior, oblique or lateral field. Customized blocks or a multi-leaf collimator will be used to shape the treatment fields. All patients will undergo a 3D planning. Beams-eye-view (BEV) displays will be used to ensure optimal target volume coverage and optimal normal tissue sparing. The most appropriate technical solutions (e.g. beam quality, field arrangement, conformal therapy planning) will be chosen as long as they comply with the International Commission on Radiation Units and Measurements (ICRU) 50/62 safety margins and homogeneity requirements.

Dose-Volume-Histograms (DVHs) of both lungs, the heart and spinal cord will be obtained for all patients. DVHs will mainly be used to document the normal tissue damage. DVHs may also help to select the most appropriate treatment plan. The volume of lung tissue that receives 20 Gy or more will not exceed 30% of the total lung volume (V20 Gy lung < 30%). The volume of the heart that receives 40 Gy will not exceed 30% of the heart volume (V40 Gy heart < 30%) and the volume of the liver that receives 30 Gy will not exceed 60% of the total liver volume (V30Gy liver < 60%). There is no limit for the maximal length of involved esophagus as long as the normal tissue criteria are not exceeded.

The risks for severe pneumonitis for patients treated under this protocol will be minimized as the volume of both lungs will be limited by the use of BEV planning and field-shaping (with optimal sparing of both lungs). The spinal cord tolerance (50 Gy) will not be exceeded with this technique.

Radiation therapy will be delivered with megavoltage equipment with photon energies of equal to or greater than 6 MV. A multileaf collimator or individually shaped blocks will be used to shape the irradiation portal according to the planning target volume.

The prescription dose will be specified at the ICRU 50/62 reference point, which will be the isocenter for most patients. The daily prescription dose will be 1.8 Gy at the ICRU reference point and the 95% isodose must encompass the entire planning target volume (PTV). The maximum to the PTV must not exceed the prescription dose by > 7% (ICRU 50/62 guidelines). Tissue density inhomogeneity correction will be used. Portal images will be obtained during the first fraction of all fields. On indication portal images will be repeated.

### Chemotherapeutic toxicity

If on day 8, 15, 22, 29, and 36 the WBC are < 1.0 × 10^9^/L and/or platelets < 50 × 10^9^/L, chemotherapy will be delayed by 1 week until recovery above these values. In case of febrile neutropenia (granulo's < 0.5 × 10^9^/L and fever > 38.5°C) or in case of severe bleeding or requiring ≥ 2 units of platelet transfusions further chemotherapy will be withheld.

Hypersensitivity reactions will be classified as mild, moderate or severe. Definitions and management guidelines are outlined in table 1. Other toxic reactions and the prescribed management of these reactions are outlined in table 2.

**Table 1:** Hypersensitivity reactions after chemotherapy: classification and management

**Classification of reactions**	**Management of reactions**
One or more mild symptoms:	Complete chemotherapy infusion with supervision at bedside. No treatment required.
• mild flushing	
• rash	
• pruritis	

One or more moderate symptoms:	Stop chemotherapy infusion, venous infusion of antihistamine (Clemastine 2 mg IV and Dexamethasone 10 mg IV), → after recovery of symptoms resume paclitaxel infusion at a rate of 20 ml/h for 15 minutes then 50 ml/h for 15 minutes then, if no further symptoms, at full dose rate until infusion is complete.
• moderate rash	
• flushing	
• mild dyspnea	
• chest discomfort	
• mild hypotension	

One or more severe symptoms:	Stop chemotherapy infusion, give IV antihistamine and steroid as above. Add epinephrine or bronchodilators if indicated, report as an adverse event, the patient will go off protocol therapy.
• respiratory distress requiring treatment	
• generalized urticaria	
• angioedema	
• hypotension requiring therapy	

**Table 2:** toxic reactions and prescribed management

**Reaction**	**Management of reactions**
Renal:	
Creatinin ≤ 1.5 × upper limit of normal at day of treatment	Continue treatment
Creatinin > 1.5 × upper limit of normal	Establish intravenous infusion the evening preciding treatment at a rate to correct any volume deficits and produce a urine flow ≥ ml/h. Repeat serum creatinin value in the morning:
	≤ 1.5 × upper limit of normal → proceed treatment
	> 1.5 × upper limit of normal → stop chemotherapy

Gastrointestinal:	
Mucositis with oral ulcers or protracted vomiting despite antiemetic premedication	Delay chemotherapy one week

Neurologic:	
CTC grade ≤ 2	Continue therapy
CTC grade > 2	Stop chemotherapy

Cardiac:	
Asympotomatic bradycardia or isolated asymptomatic ventricular extrasystoles	Continue therapy under continuous cardiac monitoring
First degree AV block	Continue therapy under continuous cardiac monitoring
Symptomatic arrhythmia or AV block (except 1^st ^degree) or other heart blocks	Stop chemotherapy, manage arrhythmia according to standard practice; patient goes off protocol

CTC Common Toxicity Criteria

### Radiation toxicity

Radiotherapy, especially concurrent with chemotherapy can lead to acute esophagitis. In some cases medical support and/or a feeding tube will be necessary. In the event of grade 4 radiation induced esophagitis both chemotherapy and radiotherapy will be withheld until the esophagitis has recovered to grade 3. Other acute complications of the radiation therapy are erythema, cough, nausea, fatigue and weight loss. In the first weeks to six months after the irradiation radiation pneumonitis or fistula formation can occur.

### Surgery

Patients randomized for surgery alone will be treated as soon as possible after randomization. In the chemoradiation arm, surgery will be performed preferably within 6 weeks after the completion of the chemoradiation. For carcinomas proximal to the tracheal bifurcation a transthoracic esophageal resection with a two field lymph node dissection is preferred. For carcinomas distal of the tracheal bifurcation but proximal to the gastro-esophageal junction, a transthoracic approach with a two field lymph node dissection or a transhiatal approach can be performed, depending on both patient characteristics and local expertise.[31] For distal tumors involving the gastro-esophageal junction a transhiatal esophageal resection is preferred.

A wide local excision including the N1 lymph nodes is carried out in both techniques including a standard excision of the lymph nodes around the celiac axis (separately collected with left gastric artery marked by a suture). The continuity of the digestive tract will be restored by a gastric tube reconstruction or a colonic interposition procedure with an anastomosis in the neck.

### Pathology

The resection specimen will be evaluated essentially using the standard protocol (margins, tumor type and extension, lymph nodes). The most recent UICC protocol is used for TNM-classification and stage grouping.[32,33] In these resection specimens special attention will be given to the effects of the neoadjuvant chemoradiation, i.e. tumor reduction and therapy effects. The effect of the chemoradiation varies from zero to 100%. In many cases a multifocal tumor appearance is present with intertwined therapy effects. In some cases only scattered tumor cells are visible, often with bizarre morphologies. Therapy effects include necrosis, inflammation with multinucleated giant cells, fibrosis and calcifications. In general fibrosis is the most remarkable effect, and it can be used to judge the extension of the untreated tumor. The Mandard score is used to quantify the anti-tumor effect.[34] The lymph node dissection should contain at least 10 nodes derived from both regional (mediastinal, esophageal) and distant sites (celiac region).

The pathology report should contain the following: site of the tumor/lesion, type and grade of the tumor, extension into the esphageal wall, resection margins, therapy effects (Mandard score), lymph node status including the site and the number of nodes with therapy effects.

### Follow-up

After completion of the protocol, patients will be followed up every 3 months for the first year, every 6 months for the second year, and then at the end of each year until 5 years after treatment, to document late toxic effects and, if applicable, disease relapse or progression, and death. Patients in the chemoradiation arm will be asked to fill out quality of life questionnaires before and after therapy and every three months during the first year of follow up. Patients randomized for surgery alone will fill out the questionnaires before surgery and every three months during the first year of follow-up.

### Statistical analysis

Data will be analyzed according to the 'Intention to treat' principle. We believe that an estimated difference in median survival of 16 months (surgery alone arm) versus 22 months (multimodality treatment arm) would justify applying this regimen in the future. We calculated that for this purpose 350 patients, 175 patients per arm, have to be enrolled. We assumed a two-sided significance level of 0.05 and a power of 0.80.[35] Survival will be dated from the date of randomization to death. Estimates of median overall survival will be based on the Kaplan-Meier method and log-rank tests will be used to determine significance. Cox regression analyses will be conducted to identify prognostic factors for survival benefit, which will be used in adjusted analyses of the treatment effect.

### Ethical considerations

The responsible physician will inform the patient about the background and present knowledge on the drugs under study with special reference to known activity and toxicity. It must be emphasized that the patient is allowed to refuse the treatment either before or at any time during the study. Before the patient is entered in the study the patient's written consent will be obtained. The principal investigator (AvdG) will ensure that this study will be carried out in agreement with the "Declaration of Helsinki, Tokyo, Venice" or the laws and regulation of the country, whichever provides greater protection of the individual. The study has been approved by the institutional ethical review committee.

### Adverse events

Any unexpected clinical adverse event or abnormal laboratory test value that is serious, including death or overdose, occurring during the course of the study, irrespective of the treatment received by the patient, must be reported to the study coordinator within one working day of occurrence.

An adverse event is serious if it is fatal or life threatening, permanently disabling, requiring hospitalization other than for planned treatment or if it is an overdose. A death occurring during treatment within 4 weeks after stopping treatment, whether treatment-related or not must be reported to the study coordinator.

## Discussion

A number of phase III trials have been performed comparing chemoradiotherapy followed by surgery versus surgery alone.[11,36-44] Only two trials showed significant benefit for use of neoadjuvant chemoradiotherapy, of which the Walsh study has been heavily criticized mainly because of poor outcome of patients treated with surgery alone, and the CALGB 9781 study because of accrual of only 56 of the 500 patients who were initially intended to be included.[41,44] In the 8 other trials no benefit for the chemoradiotherapy arm could be demonstrated.[36-40,42,43]

Several meta-analyses have been performed over the years, with varying conclusions.[15,45-49] The most recent meta-analysis from Gebski *et al*. reported a 13% absolute difference in survival at 2 years in favor of those patients receiving neoadjuvant chemoradiotherapy.[46] However, it can not be overemphasized that all these meta-analyses were based on studies with small sample sizes and that staging techniques, surgical quality and quality of chemoradiotherapy were frequently not meeting today's standard. Furthermore, a considerable heterogeneity is present between the populations of the various trials and there was an overrepresentation of patients with squamous cell carcinomas. In addition, studies using both sequential chemoradiotherapy and concurrent chemoradiotherapy were included in this meta-analysis. Finally, no correction for the extra time that chemoradiotherapy brings along for patients was made in any of the trials or meta-analyses. For instance, if chemoradiation yields a survival benefit of a few months and it also costs a few months of extra time for recovery compared with surgery alone, the benefit would be completely lost. Recapitulating, conclusions of even well performed meta-analyses based on poorly designed primary studies remain questionable.

Currently most patients in Europe and the US diagnosed with esophageal cancer present with adenocarcinomas located in the distal esophagus. Many of these patients are nowadays routinely treated with neoadjuvant chemoradiotherapy. Unfortunately, this policy is based on data of less than 200 patients with an adenocarcinoma of the esophagus treated with concurrent neoadjuvant chemoradiotherapy, as shown by the latest meta-analysis. Therefore, we believe surgery alone is still an acceptable treatment for patients with resectable esophageal cancer.

Based on the favorable results of our phase II trial with a neoadjuvant chemoradiotherapy regimen consisting of weekly administrations of Carboplatin and Paclitaxel combined with concurrent radiotherapy we initiated a phase III trial comparing this regimen with surgery alone in a sufficiently large group of patients. The accrual of this trial, a total of 350 randomized patients, is expected to be completed in the last quarter of 2008. This study will contribute to the evidence on any benefits of neoadjuvant treatment in esophageal cancer patients using a promising chemoradiotherapy regimen.

## Competing interests

The authors declare that they have no competing interests.

## Authors' contributions

MvH drafted the manuscript. AvdG and LBK co-authored the writing of the manuscript. All other authors participated in the design of the study during several meetings and are local investigators at participating centers. All authors read and approved the final manuscript.

## Pre-publication history

The pre-publication history for this paper can be accessed here:


